# Assessment of the electro-mechanical equipment’s weight of the European hydropower fleet

**DOI:** 10.1038/s41598-023-43228-1

**Published:** 2023-10-05

**Authors:** Emanuele Quaranta

**Affiliations:** European Commission, Joint Research Centre, Ispra, Italy

**Keywords:** Civil engineering, Hydroelectricity

## Abstract

Hydropower structures are typically made of materials that are available in most parts of the world, such as steel, concrete, and—to a lesser extent—copper, and do not use critical materials. The weight of hydropower structures is an important input data, as it is used to perform Life Cycle Assessments and to estimate the cost and the economic value of materials, both during the design and in case of dismantling or retrofitting. The weight of material is of interest also for policy-making purposes and for strategic development planning, for example to estimate impacts on resources. In this study, available literature equations are, for the first time, applied at a regional scale (the European Union) to estimate the weight of the hydropower fleet’s electro-mechanical (steel-made) equipment. The total weight of the electro-mechanical equipment (runner, distributor, generator, draft tube and casing) amounts to 877 ktons. The average ratio of weight to installed power is *R* = 5.7 ton/MW and it is lower in mountainous countries (*R* = 4–6 in alpine areas, *R* = 30 in Denmark), where hydropower plants exploit high heads and low discharges.

## Introduction

In 2022, hydropower accounted for 1397 GW of installed capacity, 4403 TWh/y of electricity generation, globally. In Europe, the installed power was 258 GW with a total generation of 569 TWh/y, while the installed capacity in the European Union (EU) was 155 GW, with an average annual generation of 360 TWh/y in the last decade^1,2^. The EU is the focus of this study. The EU includes: Austria, Belgium, Bulgaria, Croatia, Cyprus, Czechia, Denmark, Estonia, Finland, France, Germany, Greece, Hungary, Ireland, Italy, Latvia, Lithuania, Luxembourg, Malta, Netherlands, Poland, Portugal, Romania, Slovakia, Slovenia, Spain, Sweden. Europe also includes: Albania, Andorra, Belarus, Bosnia and Herzegovina, Faroe Islands, Gibraltar, Greenland, Iceland, Kosovo, Liechtenstein, Macedonia, Moldova, Monaco, Montenegro, Norway, San Marino, Serbia, Switzerland, Turkey, Ukraine, United Kingdom.

The overall efficiencies of hydropower plants generally exceed 80%, and can exceed 90%, with turbine’s efficiency higher than 90%^3^. Although hydropower is a well-affirmed technology, novel technologies are under development to deal with the emerging challenges of the energy market (e.g. the need or more flexibility) and to mitigate impacts on the environment^4–6^.

A hydropower facility is commonly made of a weir (or dam) to store or regulate/divert the water flow and hydraulic structures to convey water to the powerhouse (e.g., canals, tunnels, penstocks), that hosts the electro-mechanical and hydraulic equipment. Hydropower plants in water and wastewater networks, in existing barriers, and hydrokinetic turbines, generally do not require the construction of additional barriers^7^.

**Figure 1:**
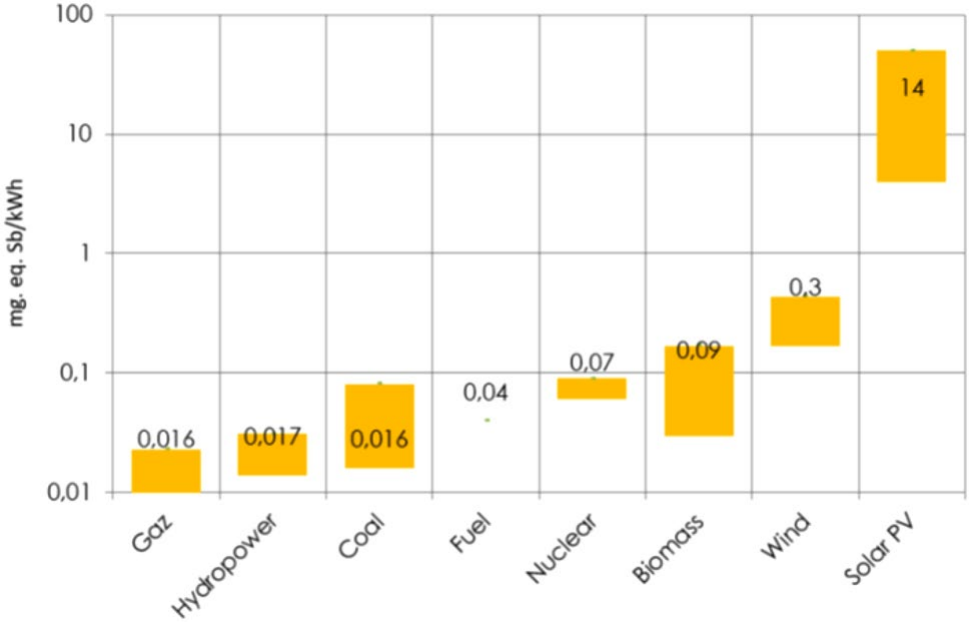
Antimony equivalent (kgeq.Sb) per energy technology^7^.

Concrete is used for dam construction and for the civil works for hydropower infrastructures, including the powerhouse. In large-scale high-head hydropower plants, concrete may also be used in the construction of intakes, tunnels and caverns. Steel is required for penstocks and steel liners of pressure tunnels and shafts, gates and valves, and for turbines. Copper is used at relatively low quantities in the generator sets. Over the last decade, new composite materials have been introduced in the hydropower sector^8^. Hydropower typically does not use critical materials, differently from photovoltaics, batteries and wind turbines. The Extraction of Mineral Resources indicator is measured in kilograms of antimony equivalent (kgeq.Sb) per kilogram extracted to take into account existing reserves, the rate of extraction and the “depletion” of each mineral substance; Fig. 1 shows this value for different energy technologies^7^.

The weight estimation of the hydropower equipment is of high relevance, both for site-specific applications and for large-scale estimations. In the former case, it allows to better estimate the amount of required material, costs and transport issues, e.g., to perform Life Cycle Assessments (LCA)^9^. The weight also affects the structural design of supporting structures (e.g.,^10^). The weight of the hydraulic machinery, together with generators, creates an important spinning reserve which allows an easier frequency control in the grid due to the energy stored in the rotating mass of the equipment^11^. Finally, during dismantling (e.g.,^12^), the knowledge of the weight of equipment allows to estimate the value of the available material and costs associated to the dismantling and transport.

Within this context, the aim of this study is to estimate the weight of the electro-mechanical equipment of the whole European Union's hydropower fleet. This large-scale estimate can be a useful input data for policy-making decisions, e.g. to estimate costs and impacts of modernization and refurbishment activities, which are key hydropower development strategies in the European Union (EU)^13^. This assessment also provides data to estimate the economic value of available materials and the possible impact on resources, supporting similar assessments in countries where hydropower is growing significantly and where large quantities of materials are needed.

## Method

**Figure 2:**
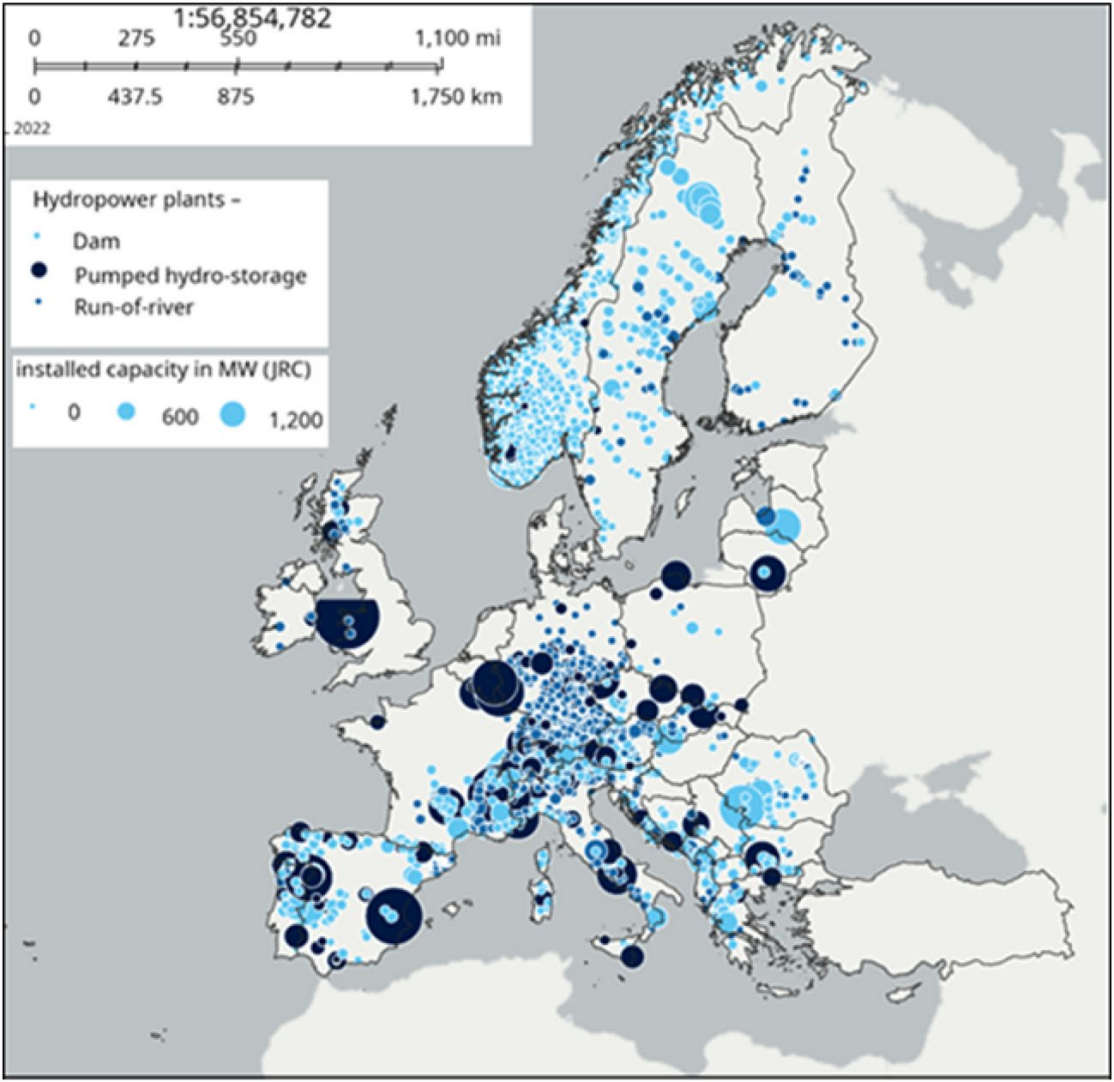
Distribution of hydropower plants in the whole Europe according to the JRC hydropower database, including 190 GW, available freely at the European Commission’s website https://energy-industry-geolab.jrc.ec.europa.eu/.

Quaranta et al.^14^ proposed a set of equations to estimate the weight of hydropower equipment. The equations, with an average absolute error which is typically below 20%, proved to be appropriate for large-scale preliminary estimates. These equations were here applied to the EU hydropower database (already described in^15^ and including turbine type, head (*H,* m), flow rate (*Q*, m^3^/s), power (*P*, MW) and number of units per plant), to estimate the weight of the electro-mechanical equipment, assuming a specific weight of 78 kN/m^3^. The cumulative installed capacity included in this database is 154 GW (158 GW including UK), which reduces to 151.6 GW when considering the power plants whose all the abovementioned data are known. In this work, the analysis was applied to the 151.6 GW of power plants with known data representing almost the whole European Union's hydropower fleet in terms of installed capacity. Finally, results were linearly extrapolated to the current installed capacity of 154 GW of the EU. The geographical distribution of the European hydropower fleet is depicted in Fig. 2.

### Kaplan, Propeller and Francis runners

The weight *G* (kN) of steel runners is a function of the turbine diameter *D* (m):

1 $$G = { 6}D^{{{2}.{75}}} \;{\text{for Francis}}$$

2 $$G = { 2}.{3}D^{{3}} \;{\text{for Propeller}}$$

3 $$G = { 3}0D^{{{1}.{9}0}} \;{\text{for Kaplan}}$$

where the diameter *D* (m) can be calculated as proposed in^16^ as a function of the rotational speed *N* (rpm). The rotational speed can be estimated as a function of the dimensionless flow rate Q*$=\frac{{Q}_{nom}}{\sqrt{2gH}{H}^{2}}$ (Eq. 4), as detailed in^17^. Under the category of Francis turbines we also included Pump-as-Turbines, Deriaz and Girard turbines.

4 $$N= \left(\alpha {Q}^{*\beta }\right)\frac{\sqrt{2gH}}{H}$$

where α = 20.3 and 26.8 for Francis and Kaplan-Bulb turbines, respectively. β = − 0.36 and − 0.38 for Francis and Kaplan-Bulb turbines, respectively. When applying Eq. (4), the same coefficients used for Kaplan turbines were also used for Bulb turbines, when the specific speed, expressed as *N *$\sqrt{P}{H}^{-1.25}$*,* was above 700. The specific weight was set to 78 kN/m^3^.

### Pelton runners

The weight of Pelton runners can be estimated by Eq. (5)

5 $$G\left( {{\text{kN}}} \right) = {439}0\;f_{1}^{2.5} \;{\text{with R}}^{{2}} = 0.{95}$$

with *f*_*1*_ = $\sqrt{\frac{Q/ {n}_{j}}{\sqrt{2 g H}}}$, where *Q* is the flow per unit (m^3^/s), *n*_*j*_ is the number of jets and *H* is the net head (m). The parameter *f*_*1*_ is an indicator of the jet diameter, and hence of the runner diameter and width. The average error of Eq. (5), estimated in^14^, was 19.8%.

### Banki runners

The following equations were used:

6 $$G = {24}.{7}\;Q^{{0.{85}}} ,{\text{ for the mechanical}}/{\text{hydraulic group}}$$

7 $$G = \, 0.{11}\;P_{el}^{{0.{98}}} ,{\text{ for the generator}}$$

where *P*_*el*_ is the electrical power in in kW and *Q* is the design flow in m^3^/s. The weight calculated by Eq. (6) includes the runner, the external casing, the inlet nozzle and supports, according to data of IREM SpA (Italy). In this study, we assumed that half of the weight is of the runner and the rest is considered as “casing”.

### Electric generator

The weight depends on the generator rotational frequency *N* (rpm) and power *P* (MVA) as per Eq. (8).

8 $$G ={ \alpha }{\left(\frac{P}{\sqrt{N}}\right)}^{\beta }$$

The average absolute error estimated in^14^ was 7.4%. For Banki turbines, Eq. (7) was used to estimate the generator weight. The values of the coefficients α and β are listed in Table 1.

**Table 1:** Coefficients of the equations for synchronous generators. Grid frequency of 50 Hz.

Number of poles	α	β
4	669	0.84
6	901	0.86
8	958	0.87
10	826	0.78
12	1024	0.80
14	1020	0.74
16	978	0.70
18	621	0.52

### Casing

The casing, normally made of steel, is a static component that encloses the rotating runner and the vanes. Its shape is rather simple for Pelton turbines, while it is spiral for reaction turbines (e.g., Kaplan and Francis turbines).

For Pelton turbines, the casing weight can be estimated by the following equations:

9 $$G = { 1177}f + {4}.{\text{92 for vertical axis }} < { 1}0{\text{ MW}}$$

10 $$G = { 763}f + { 2}.{24}\;{\text{for horizontal axis }} < { 1}0\;{\text{MW}}$$

11 $$G = { 134}.{8}f + {\text{ 99 for vertical axis }} > { 1}0\;{\text{MW}}$$

with *f* = *D*^2^ $\sqrt{\frac{Q/ {n}_{j}}{\sqrt{2 g H}}}$, *D* is the runner diameter (m), *Q* is the flow per unit (m^3^/s), *n*_*j*_ is the number of jets and *H* is the net head (m). R^2^ ranges between 0.71 and 0.94 depending on the configuration, and the average absolute error was estimated to be below 30%^14^. The vertical axis configuration was chosen when the number of jets was higher than 2. The number of jets can be estimated as:

12 $${n}_{j}= \frac{Q}{0.0039 {P}^{0.5643}}$$

where *P* (kW) and *Q* (m^3^/s) are the values per unit. For further details see^18^.

For reaction turbines, Brekke^19^ showed that the weight *G* of high head large Francis spiral casing has almost reached a stable value with the years of 30 kN/MW of installed capacity, and no further weight reduction is expected. For smaller turbines, the following equations were used^14^:

13 $$\frac{G}{P}=2.84{\left(\frac{Q}{\sqrt{H}}\right)}^{-0.81}\text{ for Vertical axis turbines}$$

14 $$\frac{G}{P}=\frac{1032}{H}\text{ for Horizontal axis turbines}$$

where *P* is the installed power capacity expressed in MW and the *G* is the weight in kN, *Q* is the flow in m^3^/s and *H* the net head in m. R^2^ = 0.8 and average absolute error was estimated to be 20%. In our analysis, we applied these equations to power plants below 10 MW, and assumed 30 kN/MW for larger power plants. According to data of Zeco Hydropower, vertical axis turbines are used below 1.25 m^3^/s (from a simple statistic, with no particular technical justification).

The weight of guide vanes was calculated considering the traditional engineering practice, as *G* = *z*∙(*l∙s*)∙*m*∙*h*∙*ρg*, where *l* is the vane length, *s* is the vane thickness (20%*l*), *m* = 0.5 is the filling ratio of the cross section (the real cross section—NACA profile—is inscribed in the rectangular area *l*∙*s*), *h* is the vane height and *z* is the number of vanes. The number of vanes can be calculated as 0.25*D*^0.5^ + 5^20^ with *D* the diameter in mm, *l* is calculated to ensure that when all the vanes are closed, the distributor diameter is completely closed (and vanes should overlap by 15%), *h* = (0.134 ln(*N*_*s*_) − 0.45)*D* with the diameter *D* in m^16^.

### Draft tube

The following empirical equations were used to estimate the weight of the draft tube for turbines below 10 MW:

15 $$\frac{G}{{N}_{s}}=0.305\, {D}^{3} \quad \text{for Horizontal axis Francis turbines with elbow draft tube}$$

16 $$\frac{G}{{D}^{3}}=16.2 \,{D}^{-1.85} \quad \text{for Vertical axis Kaplan turbines with elbow draft tube}$$

17 $$\frac{G}{{N}_{s}}=0.0205\, {D}^{1.95} \quad \text{for Propeller turbines}$$

The elbow draft tube, which is the most used and efficient type, was assumed for Francis and Kaplan turbines, while Propeller turbines use straight draft tubes^14^. R^2^ > 0.82 and the estimated average absolute error was 20%. *N*_*s*_ is the specific speed expressed as *N* $\sqrt{P}{H}^{-1.25}$, with *P* in kW, the net head *H* in m and *N* in rpm. *D* is the draft tube inlet diameter in m, calculated by equations proposed in^16^. For Kaplan turbine units with elbow and S-type draft tube, the weight estimated by Eq. (16) only includes the first part of the draft tube, because the second (diffuser) part is typically made of concrete built on-site. These equations are considered valid also for power plants above 10 MW.

### Steel in the powerhouse structure

The steel used in the powerhouse can be estimated according to^21^, in KN, as:

18 $$G=k (n+R){C}^{0.358 }{D}^{1.074}$$

where *k* = 32.5 and 79.6 for reaction and impulse turbines, respectively. *C* = Crane capacity in tons, *D* = Runner throat diameter in meters, *n* = Number of units in powerhouse, *R* = Repair bay ratio, that can be assumed as equal to 0.5^14^. The Crane capacity can be estimated as *G* = 46 ${\left(\frac{P}{\sqrt{N}}\right)}^{0.8}$^22^, similarly to Eq. (8). This value does not belong to the electro-mechanical equipment.

## Results and discussion

**Figure 3:**
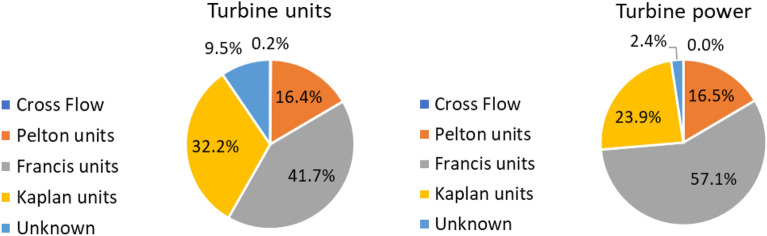
Share of turbine type.

Figure 3 shows the share of turbine types, based on the installed power and number of units. The Francis type, being the one with the broadest application range, is the most widely used. 24 Cross-Flow Banki turbines are present in the database.

The equations were applied to the available dataset, and Table 2 shows the estimated equipment’s weight for each country, together with the considered installed power (available in the database) and the total real installed power. Table 3 shows the same results but disaggregated for each equipment component. The average elevation on the sea level of each country is also included, as mountainous countries in the EU (to which higher elevations are associated) typically host a higher hydropower potential (Fig. 2). The total weight is 862 ktons, associated to 151.6 GW of installed power. If results are linearly extrapolated to the 153.7 GW, 877 ktons is obtained. The draft tube contributes by 39% of this (it is the heaviest component), the generator by 22%, the casing by 17%, the runner by 15% and the guide vanes by 7%. The weight of generators is higher than the runner one. Also the cost of generators is generally higher than the cost of runners^23^. The weight of steel in the powerhouse was analysed separately as it is not part of the electro-mechanical equipment. The steel in the powerhouse amount to 963 ktons at the EU scale; the ratio of the weight of the powerhouse (the steel amount) to the equipment's weight, at the member state level, ranges between 0.55 and 3.18, with an average ratio of 1.28.

**Table 2:** Estimated values per country.

EU country	Considered power *P* (MW)	Total power (MW)	Weight *G* (tons)	*G*/*P*	avg. country elevation on the sea level (m)	Superstructure powerhouse steel (tons)
Austria	14,186	14,312	70,047	4.94	910	104,000
Belgium	1291	1304	5120	3.97	181	6122
Bulgaria	3009	3177	9265	3.08	470	7522
Croatia	2195	2201	7875	3.59	331	7711
Cyprus	1	1	14	16.50	91	13
Czech Republic	1987	2003	12,084	6.08	430	15,232
Denmark	2	20	47	30.25	34	70
Estonia	3	3	65	25.38	61	92
Finland	2700	3264	21,102	7.82	164	42,277
France	25,525	27,001	111,490	4.37	375	159,647
Germany	10,209	10,690	142,073	13.92	263	126,112
Greece	3293	3306	13,722	4.17	498	12,868
Hungary	66	70	1697	25.84	143	2419
Ireland	540	544	3102	5.75	118	3177
Italy	23,050	23,974	90,868	3.94	538	73,671
Latvia	1555	1555	20,209	13.00	87	17,321
Lithuania	825	934	2028	2.46	110	2296
Luxembourg	1298	1302	4311	3.32	325	2862
Malta	NA	NA	0	NA	NA	
Netherlands	43	43	864	20.18	30	2744
Poland	2395	2406	18,955	7.91	173	22,719
Portugal	7448	7616	28,810	3.87	372	32,871
Romania	6924	7160	36,317	5.25	414	60,431
Slovakia	2439	2440	11,865	4.87	458	21,748
Slovenia	1046	1239	5643	5.40	492	12,098
Spain	19,015	20,360	163,314	8.59	660	89,063
Sweden	16,212	16,811	84,258	5.20	320	126,885
Total EU	151,617	153,734	865,142	5.8		962,644

In the weight column, only the power plants with known power, head, flow per unit and turbine type were considered, for an installed power indicated in the column “Considered Power”.

*NA* not available.

**Table 3:** Estimated values per country.

EU country	Considered power *P* (MW)	Total power (MW)	Runner (tons)	Casing (tons)	Draft tube (tons)	Generator (tons)	Guide vanes (tons)
Austria	14,186	14,312	17,074	16,130	9400	18,971	8472
Belgium	1291	1304	616	1854	1607	679	365
Bulgaria	3009	3177	574	2008	738	5807	138
Croatia	2195	2201	773	973	1325	4470	333
Cyprus	1	1	0	2	2	9	0
Czech Republic	1987	2003	1798	2565	4348	2114	1258
Denmark	2	20	16	23	5	0	4
Estonia	3	3	10	23	18	12	2
Finland	2700	3264	7640	3869	5847	165	3581
France	25,525	27,001	20,163	19,230	30,233	32,155	9708
Germany	10,209	10,690	19,493	33,197	63,532	15,660	10,397
Greece	3293	3306	1011	1769	8964	1666	312
Hungary	66	70	297	907	224	79	190
Ireland	540	544	377	363	1130	1104	127
Italy	23,050	23,974	7396	20,645	10,624	49,699	2629
Latvia	1555	1555	2638	504	15,506	14	1546
Lithuania	825	934	145	299	1543	0	41
Luxembourg	1298	1302	125	711	438	2957	80
Malta	NA	NA	0	0	0	0	0
Netherlands	43	43	325	15	127	16	381
Poland	2395	2406	2703	4281	8534	2051	1387
Portugal	7448	7616	3599	3700	15,200	4689	1621
Romania	6924	7160	9648	8173	4262	10,070	4335
Slovakia	2439	2440	3361	2328	2190	2442	1544
Slovenia	1046	1239	2104	793	916	918	912
Spain	19,015	20,360	8264	15,313	105,736	30,818	3183
Sweden	16,212	16,811	18,235	11,181	42,938	3523	8380
Total EU	151,617	153,734	128,385	150,856	335,385	190,088	60,930

In the weight column, only the power plants with known power, head, flow per unit and turbine type were considered, for an installed power indicated in the column “Considered Power”.

**Figure 4:**
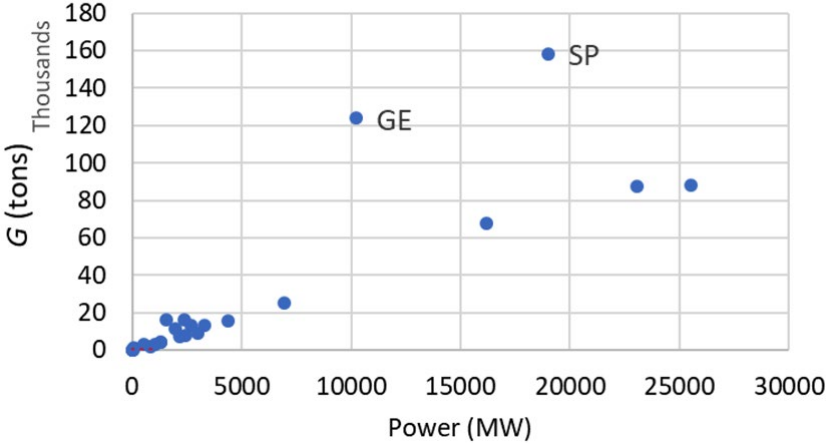
Weight versus installed power. GE = Germany, SP = Spain.

**Figure 5:**
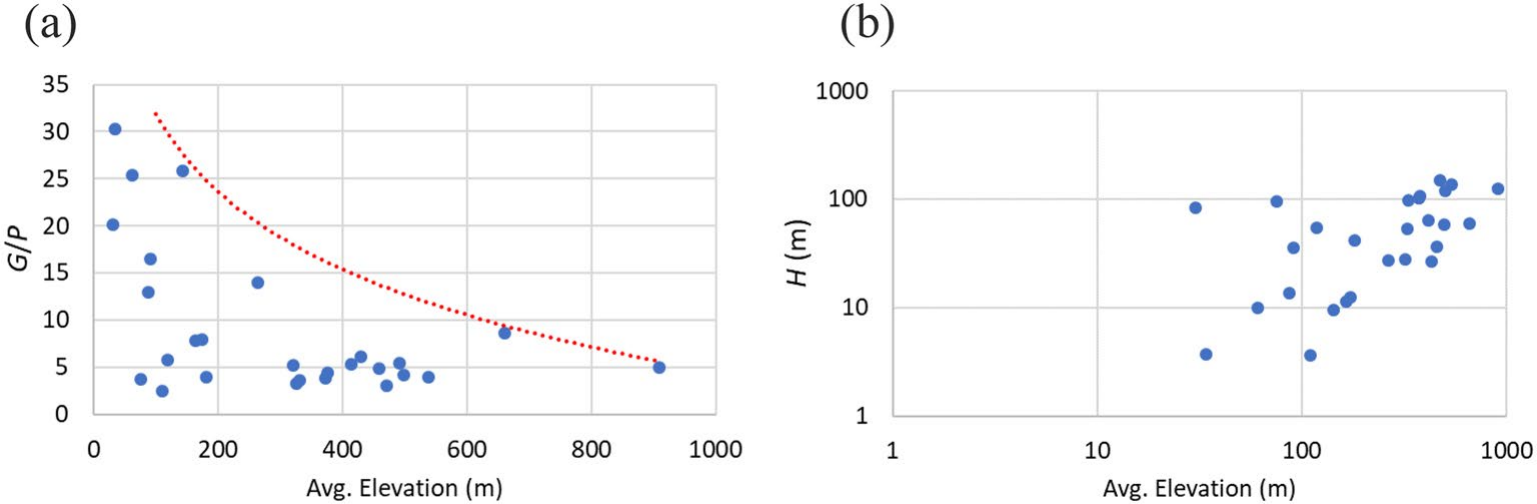
Ratio *G*/*P* versus country average elevation (**a**); average head versus country average elevation; each point is a country (**b**).

**Figure 6:**
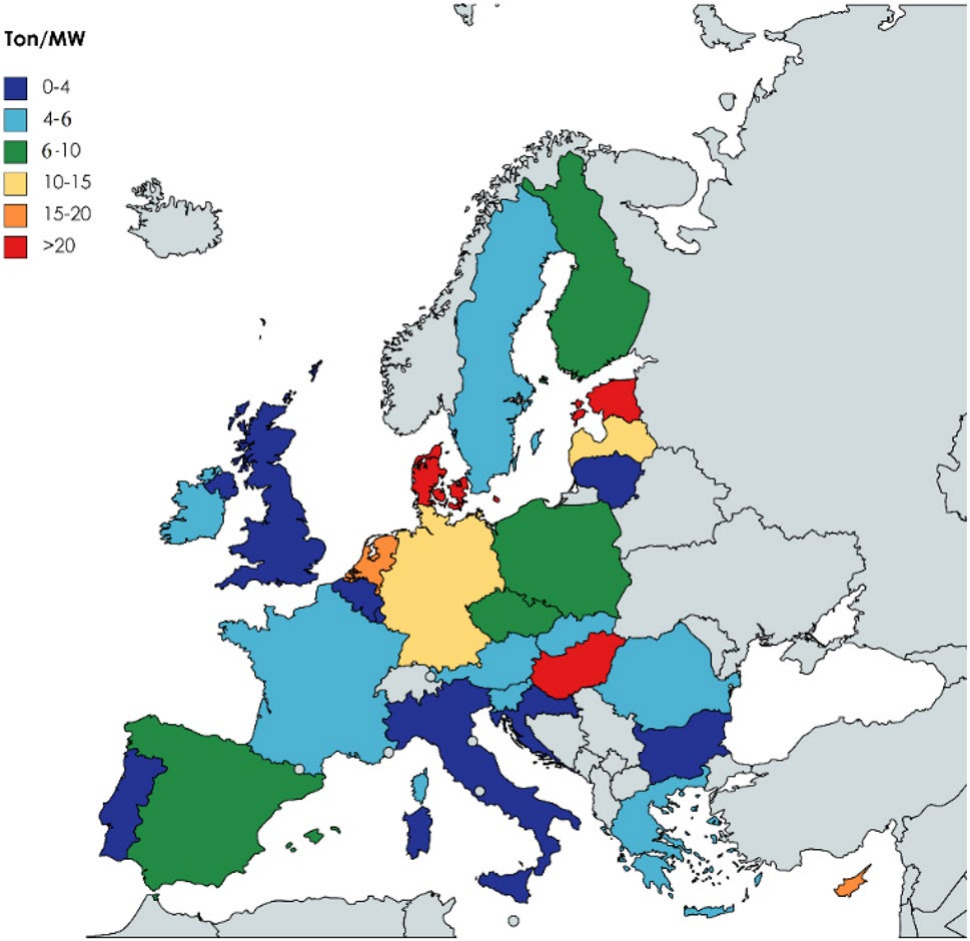
Ratio *G*/*P* in each member state. Map generated using the open source website https://www.mapchart.net/europe.html.

Figure 4 shows that the weight increases with the installed power (for each member state) with an almost linear trend, and the weight is much higher in Germany and Spain: this may be due to their larger share of Francis turbines (74%) and lower share of Pelton turbines (5.6%) with respect to average EU values. Figure 5a show the ratio *G*/*P* for each country versus the average altitude on the sea level. The ratio *G*/*P* reduces with the average country elevation, because high head power plants, that typically exploit higher heads and lower discharges (Fig. 5b), are smaller and lighter. Figure 6 shows the EU map with the ratio *G*/*P*. The turbines employed for high-head applications must be made with materials capable of resisting both the high stresses generated by the water pressure and fatigue, erosion and cavitation. Low-head turbines do not experience high stresses and pressures, but the weight/power ratio is quite high.

In the analysis, some simplifications had to be implemented. The equations for Francis turbines were also applied to Deriaz, Girard and Pump turbines, based on the assumption that the geometric appearance of these turbines is similar. The study estimated the weight of the runner, distributor, generator, casing and draft tube, but did not include gates, nozzles (e.g., in Pelton turbines), auxiliary steel equipment (shafts, supports, connectors, trunnions) and penstocks, that are very site-specific. The equations for reaction turbines were developed for runner diameters above 0.8 m, while the runners of 43% of power plants in the database have an estimated diameter below 0.8 m.

Most of mini hydropower plants (< 1 MW) are not covered by the database; nevertheless, power plants below 1 MW represent 3 GW of the EU hydropower fleet (1.9%)^24^, of which a negligible share is represented by unconventional turbines, e.g. water wheels, Archimedes screws and Very Low Head (VLH) turbines. The installed capacity of Archimedes screws is typically below 100 kW and in 2012 around 400 installations were counted worldwide, 71 of them in Europe, for a total European installed power of 2.5 MW^25^. VLH turbines are typically below 500 kW and their total installed capacity in Europe is around 30 MW^26^ (they were probably considered in the database as “Kaplan” type). Water wheels are typically below 30 kW and with average power of 13 kW in the EU^24^, and it might be possible to assume that their cumulative installed capacity in the EU is below 10 MW. In the EU, 12 Vortex turbines are in operation, with a cumulative power of 78.1 kW (in Austria, Germany and Belgium)^27^. Therefore, unconventional turbines represent less than 50 MW in the EU and affect the overall weight to a less extent.

In its exploratory intent, this calculation provides a first estimate of the hydropower fleet’s weight in the EU, with focus on the steel-equipment. Future researches should aim at providing more expeditious tools and data to estimate better the weight of pico- and micro-turbines and the weight of large draft tubes. Similar analyses should be carried out also for the other energy technologies. In the EU, future trends will mainly consist in modernisation, digitalisation and sustainability increase of hydropower plants, so that the main issue is associated to steel rather than to cement and concrete. However, it must be noted that, especially for the countries where hydropower is expanding significantly, concrete depends on sand availability. Sand is the second-most used resource on Earth, after water. It is often dredged from rivers, dug up along coastlines and mined. This aspect should be taken into consideration.

## Conclusions

The weight of hydropower equipment is of high interest for numerous purposes. When considering large-scale purposes, knowing the weight would allow to estimate the economic value of the available material and the eventual impact on resources. In this contribution, we estimated that the electro-mechanical equipment of the European Union’s hydropower fleet weights 877 ktons, and approximately 40% of it is represented by the draft tube. Further researches should aim at providing equations to estimate the draft tube of large units (above 10 MW) and the weight of pico- and micro-turbines. Similar analyses should be performed for the other energy technologies, which, contrary to the hydropower sector, often use critical materials and rare earths. More analyses of the steel recycling and reuse process in the context of electro-mechanical equipment in hydropower plants should be carried out. In addition, an analysis of production-related costs and potential savings from efficient management of steel resources could add value. Identification of possible alternative materials that could replace steel in certain aspects of construction would also be valuable.

## Data Availability

The datasets used and/or analysed during the current study are available from the corresponding author on reasonable request.

## List of symbols

*D*: Runner diameter (m)
*d*: Jet diameter (m)
*f*: Dimensionless parameter
*g*: Acceleration of gravity (m/s^2^)
*G*: Self-weight force applied in the gravity center of the mass considered (kN)
*H*: Net head (m)
*i*: Number of units
*L*: Length (m)
*N*: Rotational speed (rpm)
*n*_*j*_: Number of jets
*P*: Power (kW)
*Q*: Flow rate (m^3^/s)
*ρ*: Material density (kg/m^3^)
